# SWIPT-Aware Fog Information Processing: Local Computing vs. Fog Offloading

**DOI:** 10.3390/s18103291

**Published:** 2018-09-30

**Authors:** Haina Zheng, Ke Xiong, Pingyi Fan, Li Zhou, Zhangdui Zhong

**Affiliations:** 1 School of Computer and Information Technology, Beijing Jiaotong University, Beijing 100044, China; hnzheng@bjtu.edu.cn; 2 Department of Electronic Engineering, Tsinghua University, Beijing 100084, China; fpy@tsinghua.edu.cn; 3 School of Information, Beijing Wuzi University, Beijing 101149, China; zhoulibit@126.com; 4 State Key Lab of Rail Traffic Control and Safety, Beijing Jiaotong University, Beijing 100044, China; zhdzhong@bjtu.edu.cn

**Keywords:** energy harvesting, simultaneous wireless information and power transfer, fog computing, local computing, fog offloading, deployment scheme

## Abstract

This paper studies a simultaneous wireless information and power transfer (SWIPT)-aware fog computing by using a simple model, where a sensor harvests energy and receives information from a hybrid access point (HAP) through power splitting (PS) receiver architecture. Two information processing modes, local computing and fog offloading modes are investigated. For such a system, two optimization problems are formulated to minimize the sensor’s required power for the two modes under the information rate and energy harvesting constraints by jointly optimizing the time assignment and the transmit power, as well as the PS ratio. The closed-form and semi-closed-form solutions to the proposed optimization problems are derived based on convex optimization theory. Simulation results show that neither mode is always superior to the other one. It also shows that when the number of logic operations per bit associated with local computing is less than a certain value, the local computing mode is a better choice; otherwise, the fog offloading mode should be selected. In addition, the mode selection associated with the positions of the user for fixed HAP and fog server (FS) is also discussed.

## 1. Introduction

With the rapid development of the Internet of Things (IoT), a growing number of sensor nodes are required to access wireless networks and arousing a large number of computation-intensive and latency-sensitive applications [1,2,3], which brings crucial challenges to resource-constraint devices. How to enhance their processing capacities has attracted great interest in both academia and industry. To resolve the related issues, fog computing (which is similar to mobile edge computing (MEC) [4,5,6]) has emerged to be a promising solution by offloading the task to nearby devices with high computing capacities [7,8,9,10,11].

In IoT systems, most devices are powered by batteries with limited energy capacities. To prolong the lifetime of the energy-constrained devices and networks (e.g., wireless sensor networks (WSN) [12], wireless personal area networks (WPANs) [13], etc.), energy harvesting (EH) has been regarded as a very promising technology, as it is able to power the devices via ambient energy sources [14,15]. In the EH family, radio frequency (RF)-based EH is one of the most popular members due to its capabilities in providing controllable and sustainable power supply. As signals also carry information when they deliver energy, simultaneous wireless information and power transfer (SWIPT) was proposed. Later, two practical receiver architectures, i.e., time switching (TS) and power splitting (PS), were proposed in Varshney et al. [16]. So far, both TS and PS have been widely applied in various wireless systems (see e.g., [17,18,19,20,21,22,23,24,25,26,27,28,29]).

Owing to the advantages of fog computing and SWIPT, inheriting their benefits is expected to provide an efficient way to simultaneously enhance the computing capacity and prolong the lifetime of energy constrained networks. So far, some works have studied the SWIPT-aware fog/MEC systems [30,31,32]. In Janatian et al. [30], the authors studied the optimal resource allocation in ultra-low power fog-computing SWIPT-based networks, where, however, only the TS receiver architecture was adopted. In Di et al. [31], the authors studied the fog-assisted resource allocation for two-hop SWIPT orthogonal frequency division multiplexing (OFDM) networks. Although the PS receiver architecture was considered in Di et al. [31], the computing task offoading was not involved. In Chai et al. [32], the power minimization problem was studied in a SWIPT-aided fog computing networks is considered. Although both PS receiver architecture and offloading were considered, the FS was just used to assign tasks rather than participating in the computing task. *To the best of the authors’ knowledge, no work has been done for the SWIPT-aware fog aided work with PS architecture, where fog offloading and local computing are jointly designed.*

Motivated by this, we focus on a SWIPT-aware fog computing system with PS receiver architecture. Compared with traditional fog computing systems or SWIPT-aware systems, our considered PS SWIPT-aware fog computing system can simultaneously enhance the computing capacity and prolong the lifetime of energy constrained networks, which is expected to be an efficient way to inherit the benefits of the fog computing and SWIPT. Nevertheless, optimally designing such a SWIPT-aware fog computing system faces some challenges, since to fully explore the potential performance of the system, the communication, the computation, and the energy resources have to be efficiently utilized together. As these resources are coupled together, which is difficult to handle, to this end, we study a three-node system model, where a sensor harvests energy and receives information from a HAP through PS receiver architecture. The sensor is able to process the received information itself (local computing mode) or offload the task to the nearby FS (fog offloading mode) with the harvested energy. For such a model, we desire to theoretically derive the inner relationships among the different parameters associated with different kind of resources, and some fundamental questions are going to be answered, e.g.,

- Is there a mode always superior to another between local computing and fog offloading?
- Which one is the better choice for a given set of system parameters?
- For a fixed mode, what is its optimal resource allocation?

To this end, two power-minimization optimization problems are formulated for the two modes under the required data rate and energy harvesting constraints by jointly optimizing the time assignment and the transmit power at the sensor, as well as the PS ratio at first. Since the problems are difficult to tackle, we solve them by using some mathematical operations and the convex optimization theory, and then the closed-form and semi-closed-form solutions to the optimization problems are derived. Simulation results show that neither of the two modes is always superior to the other one. It is also shown that when the number of logic operations per bit associated with local computing is less than a certain value, the local computing mode is a better choice; otherwise, the fog offloading mode should be selected. In addition, the mode selection associated with the positions of the sensor for fixed HAP and FS is also discussed, which shows that when the sensor is close to the HAP or the FS, the fog offloading mode is a better choice, but for the rest of positions, local computing should be selected in order to achieve a lower energy requirement at the sensor.

The rest of the paper is organized as follows. In Section 2, the system model is described. In Section 3, two optimization problems are formulated and solved for the two modes. Simulation results are provided in Section 4. Finally, conclusions are given in Section 5.

For readers’ convenience, we first summarize some notations in Table 1.

## 2. System Model

**Figure 1:**
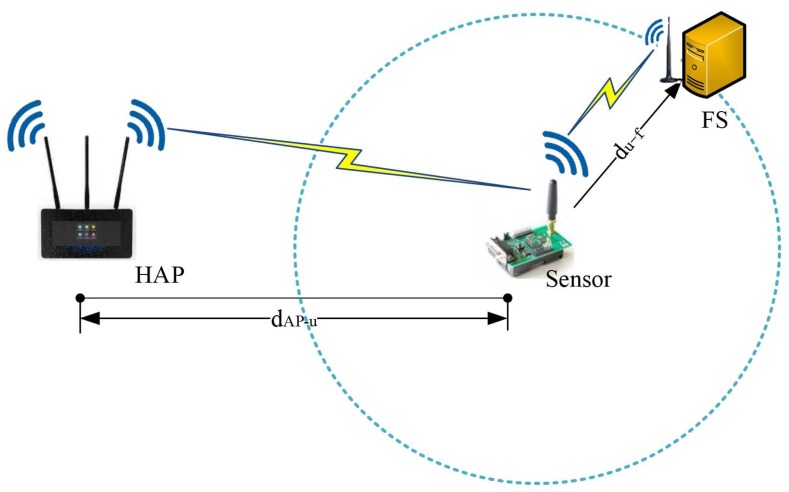
Illustration of the system model.

Consider a SWIPT-aware fog computing model consisting of a multi-antenna hybrid access point (HAP), a single-antenna sensor and a multi-antenna fog server (FS), as shown in Figure 1, where the HAP desires to transmit data to the sensor and the sensor has to process the received data by itself or by the nearby FS. Note that three-node system model is widely investigated for communication system design since it is a basic component of complex networks, which can be extended to the multiple sensors scenarios by employing time division multiple access (TDMA), frequency division multiple access (FDMA) or code division multiple access (CDMA) [33].

It is assumed that the HAP is with sufficient power supply and the sensor node is with no energy, so the HAP is used as a power source to charge the sensor. The FS is with strong computing capacity, which is capable of helping compute the tasks offloaded by the sensor and feedback the calculated result to the sensor. PS SWIPT receiver architecture is employed at the sensor, so that the sensor can harvest energy and receive information from the same signals transmitted by the HAP. The FS can be the free computation resource nearby the sensor, and also can be integrated in the HAP. It is also assumed that the sensor has some computing capacity, so the computing task can be accomplished either by the sensor itself (i.e., local computing mode) or helped by the FS (i.e., fog offloading mode). If the sensor prefers the FS to help complete the computing task, it needs to offload the data to the FS over the wireless link. Suppose the sensor knows the channel coefficients of the two links, as well as the computing capacity of the FS. Thus, it can determine which mode is a better choice. Note that whether local computing or fog offloading is selected, energy is required to perform the related computing or transmission.

**Figure 2:**
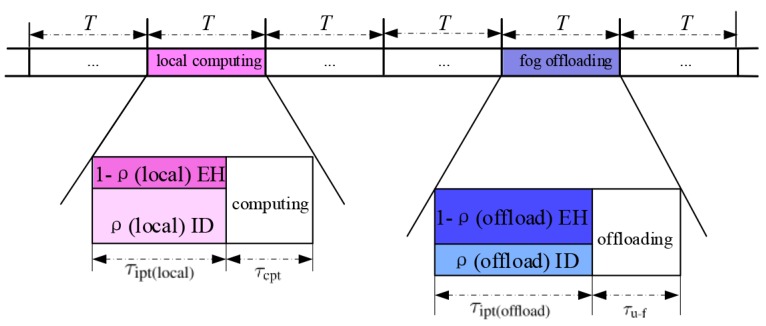
Illustration of the time frame structure.

Let *T* denote the length of each transmission frame. To complete the SWIPT-aware local computing or fog offloading, *T* is divided into two parts as shown in Figure 2. For both modes, in the first part with interval $\tau_{\mathrm{ipt}}$, the sensor harvests energy and decodes the received data from the transmitted signals by HAP, and in the rest *T*-$\tau_{\mathrm{ipt}}$, sensor processes the received information either by local computing mode or by fog offloading mode.

The complex channel vector from the HAP to the sensor is denoted with $h_{\mathrm{AP}-u}\in C^{N_{A}\times 1}$. Block fading channel model is considered, where the channel coefficient is assumed to be constant in each block and changes independently following Rician distribution from one block to the next. Without loss of generality, the time interval of each block is also represented by *T*. Perfect channel state information (CSI) is assumed in order to explore the system performance limits. Denote the number of antennas at the HAP as $N_{A}$ with $N_{A}>1$ and the RF signal symbol transmitted by the HAP as *s*, which can be originated from independent Gaussian codebooks, i.e., s∼CN(0,1), and the beamforming vector is expressed by $w\in C^{N_{A}\times 1}$. Then, the received signal at the sensor is given by

(1) $$\begin{array}{c} y=\sqrt{P_{\mathrm{AP}}}h_{\mathrm{AP}-u}^{H}ws+n, \end{array}$$

where $P_{\mathrm{AP}}$ is the transmit power of the HAP and n∼CN (0, $\sigma_{n}^{2}$) is the noise received at the receiver, which obeys the circularly symmetric complex Gaussian distribution. Since the channel between the HAP and the sensor is a multiple input single output (MISO) channel, by using the maximum rate transmission (MRT) strategy, the optimal w related to $h_{\mathrm{AP}-u}$ can be given by Xiong et al. [34]

(2) $$\begin{array}{c} w^{*}=\frac{h_{\mathrm{AP}-u}}{∥h_{\mathrm{AP}-u}∥}. \end{array}$$

With PS SWIPT receiver architecture, a part of the received signals’ power is input into the EH circuit for energy harvesting and the rest of the signals’ power at the sensor is input into the information decoding (ID) circuit for information decoding. Let ρ ∈ (0, 1) be the power splitting factor. The harvested energy at the sensor can be given by

(3) $$\begin{array}{c} E_{\mathrm{eh}}=\eta \left(1-\rho \right)P_{\mathrm{AP}}{\left|h_{\mathrm{AP}-u}^{H}w\right|}^{2}\tau_{\mathrm{ipt}}, \end{array}$$

where η∈(0,1) denotes the energy conversion efficiency of the EH circuit, and the achievable information rate $R_{\mathrm{AP}-u}$ (bits/sec) at the sensor can be given by

(4) $$\begin{array}{c} R_{\mathrm{AP}-u}=B\frac{\tau_{\mathrm{ipt}}}{T}\log\left(1+\frac{\rho P_{\mathrm{AP}}{\left|h_{\mathrm{AP}-u}^{H}w\right|}^{2}}{\sigma_{n}^{2}}\right), \end{array}$$

where *B* is the system frequency bandwidth. Following Meraji et al. [35], the required energy for information decoding at the sensor is proportional to the received information amount. Therefore, the required energy for information decoding at the sensor can be given by

(5) $$\begin{array}{c} E_{\mathrm{id}}=\xi R_{\mathrm{AP}-u}T=\xi B\log\left(1+\frac{\rho P_{\mathrm{AP}}{\left|h_{\mathrm{AP}-u}^{H}w\right|}^{2}}{\sigma_{n}^{2}}\right)\tau_{\mathrm{ipt}}, \end{array}$$

where ξ (Joule/bit) is constant, which is used to characterize the energy requirement for decoding per bit.

### 2.1. Local Computing Mode

Once the local computing mode is selected, in the second time interval, i.e., $\tau_{\mathrm{cpt}}$, the sensor processes the received data itself. To do so, energy is required for computation operations at the sensor. As described in Rabaey et al. [36], the minimum dynamic switching energy per logic gate can be evaluated by $C_{g}V_{DD}^{2}$, where $C_{g}$ is the gate input capacitance and $V_{DD}^{2}$ is the supply voltage. In this case, the energy requirements are larger than the Landauer limit by a factor of $M_{c}$, i.e., $M_{c}N_{0}\ln\;2$, where $M_{c}$ is a time-dependent immaturity factor of the technology and $N_{0}$ is the thermal noise spectral density. Thus, the local computing energy requirement at the sensor can be expressed by

(6) $$\begin{array}{c} E_{\mathrm{cpt}}=F_{0}\alpha M_{c}N_{0}\ln\;2KR_{\mathrm{AP}-u}T, \end{array}$$

where $F_{0}$ is the fanout, i.e., the number of loading logic gates (typically with value of 3 to 4), α is the activity factor (typically with value of 0.1 to 0.2), and *K* is the number of logic operations per bit. $R_{\mathrm{AP}-u}T$ actually represents the number of received bits in each *T*.

The total required energy at the sensor is described by

(7) $$\begin{array}{c} E_{u}=E_{\mathrm{id}}+E_{\mathrm{cpt}}-E_{\mathrm{eh}}. \end{array}$$

When $E_{u}\geq 0$, it means that the harvested energy is less than the total required energy $E_{\mathrm{id}}+E_{\mathrm{cpt}}$. In this case, the battery has to discharge a certain amount of energy, i.e., $E_{u}$, to help accomplish the local computing. When $E_{u}<0$, the harvested energy is more than the total required energy $E_{\mathrm{id}}+E_{\mathrm{cpt}}$, and the local computing is triggered.

### 2.2. Fog Offloading Mode

If the fog offloading mode is selected, the sensor transmits the decoded task to the FS to process. Let $h_{u-f}$ be the complex-valued channel coefficient from the sensor to the FS. The achievable information rate $R_{u-f}$ (bits/s) associated with the offloading over *T* can be given by

(8) $$\begin{array}{c} R_{u-f}=B\frac{\tau_{u-f}}{T}\log\left(1+\frac{{\left|h_{u-f}\right|}^{2}P_{u-f}}{\sigma_{s}^{2}}\right), \end{array}$$

where $P_{u-f}$ denotes the transmit power at the sensor, $\sigma_{s}^{2}$ is the receiver’s noise power, and $\tau_{u-f}$ is the time used for task offloading from the sensor to the FS. Similar to many existing works, see e.g., [28,29], we also assume that the fog server has very strong computing ability and high transmit power, so the time used for fog computing and that transmitting from the FS to the sensor could be neglected. The energy required for task offloading at the sensor is given by

(9) $$\begin{array}{c} E_{u-f}=P_{u-f}\tau_{u-f}, \end{array}$$

where $P_{u-f}$ can be adjusted and it must be constrained by the maximal available transmit power $P_{u-f}^{\left(\max\right)}$,

(10) $$\begin{array}{c} P_{u-f}\leq P_{u-f}^{\left(\max\right)}. \end{array}$$

The total required energy at the MU by using fog offloading mode is

(11) $$\begin{array}{c} E_{u}=E_{\mathrm{id}}+E_{u-f}-E_{\mathrm{eh}}. \end{array}$$

Similar to the local computing mode, when $E_{u}\geq 0$, the battery has to discharge a certain amount of power, i.e., $E_{u}$, to help accomplish the fog offloading. When $E_{u}<0$, the fog offloading is triggered.

## 3. Problem Formulation and Solution

This section formulates two optimization problems for the two modes to minimize the power requirement while guaranteeing the minimal required information rate by jointly optimizing the time assignment, the power splitting ratio and the transmit power adjustment at the sensor. Assuming that the sensor’s battery capacity is sufficient to trigger the computing or the transmission and has enough energy for completing the whole process.

### 3.1. Optimization of the Local Computing

For local computing mode, the power minimization problem can be mathematically expressed by

(12)P1:minτipt,τcpt,ρ(local)Eid+Ecpt−Eeh,s.t.C1:RAP−u≥Rth,C2:τcptfop≥KRAP−uT,C3:τipt+τcpt≤T,C4:τipt,τcpt∈(0,T),ρ(local)∈(0,1),

where $R_{\mathrm{th}}$ and $f_{\mathrm{op}}$ are the minimum information transmission rate requirement from the HAP to the sensor and the maximum number of the operations per second at the sensor, respectively. Constraint (**C2**) describes that the total number of logic operations at the sensor must be equal or larger than the minimal required operations of the task. To solve Problem $P_{1}$, we expand the expressions of variables of Problem $P_{1}$ as

(13)P1−A:minτipt,τcpt,ρ(local)BC1τiptlog1+ρ(local)PAPhAP−uHw2σn2−η1−ρ(local)PAPhAP−uHw2τipt,s.t.C1:BτiptTlog1+ρ(local)PAPhAP−uHw2σn2≥Rth,C2:τcptfop≥KτiptBlog1+ρ(local)PAPhAP−uHw2σn2,C3:τcpt≤T−τipt,C4:τipt,τcpt∈(0,T),ρ(local)∈(0,1),

where $C_{1}=KF_{0}\alpha M_{c}N_{0}\ln\left(2\right)+\xi$. Variables $\tau_{\mathrm{ipt}}$ and $\rho_{\left(\mathrm{local}\right)}$ are coupled together, so that Problem $P_{1-A}$ is non-convex and cannot be directly solved by using known solution methods. Hence, a new slack variable $\varphi =\rho_{\left(\mathrm{local}\right)}\tau_{\mathrm{ipt}}$ is introduced to make the problem more tractable. Therefore, $\rho_{\left(\mathrm{local}\right)}=\frac{\varphi }{\tau_{\mathrm{ipt}}}$ and Problem $P_{1-A}$ can be transformed to be

(14)P1−B:minτipt,φBC1τiptlog1+φPAPhAP−uHw2τiptσn2−ητipt−φPAPhAP−uHw2,(15)s.t.C1:Bτiptlog1+φPAPhAP−uHw2τiptσn2≥RthT,(16)C2:Bτiptlog1+φPAPhAP−uHw2τiptσn2≤T−τiptfopK,C3:τipt∈(0,T),φ∈(0,T).

Let $C_{2}=\eta P_{\mathrm{AP}}{\left|h_{\mathrm{AP}-u}^{H}w\right|}^{2}$, $f\left(\tau_{\mathrm{ipt}},\varphi \right)=B\tau_{\mathrm{ipt}}\log\left(1+\frac{\varphi P_{\mathrm{AP}}{\left|h_{\mathrm{AP}-u}^{H}w\right|}^{2}}{\tau_{\mathrm{ipt}}\sigma_{n}^{2}}\right)$ and $F(\tau_{\mathrm{ipt}},\varphi )$ = $BC_{1}\tau_{\mathrm{ipt}}\;\log\left(1+\frac{\varphi P_{\mathrm{AP}}{\left|h_{\mathrm{AP}-u}^{H}w\right|}^{2}}{\tau_{\mathrm{ipt}}\sigma_{n}^{2}}\right)-\eta \left(\tau_{\mathrm{ipt}}-\varphi \right)$ $P_{\mathrm{AP}}{\left|h_{\mathrm{AP}-u}^{H}w\right|}^{2}$, Problem $P_{1-B}$ can be rewritten as

(17)P1−C:minτipt,φF(τipt,φ)=C1f(τipt,φ)−C2(τipt−φ),(18)s.t.C1:f(τipt,φ)≥RthT,(19)C2:f(τipt,φ)≤T−τiptfopK,C3:τipt∈(0,T),φ∈(0,T).

The first term of $F(\tau_{\mathrm{ipt}},\varphi )$, i.e., $f(\tau_{\mathrm{ipt}},\varphi )$, is with the perspective function form of $y\log\left(1+\frac{x}{y}\right)$ which is concave w.r.t *x* and *y* [37], so, $f(\tau_{\mathrm{ipt}},\varphi )$ is concave w.r.t $\tau_{\mathrm{ipt}}$ and φ, and in the second term of $F(\tau_{\mathrm{ipt}},\varphi )$, i.e., $-C_{2}\left(\tau_{\mathrm{ipt}}-\varphi \right)=-C_{2}\tau_{\mathrm{ipt}}+C_{2}\varphi$, is linear w.r.t $\tau_{\mathrm{ipt}}$ and φ. Therefore, the objective of Problem $P_{1-C}$ is a minimization of a concave function w.r.t $\tau_{\mathrm{ipt}}$ and φ. Nevertheless, $P_{1-C}$ is still a non-convex problem also because of the non-convexity of Constraint (19). Hence, we analyze and solve it as follows:

**Proposition** **1.** *Problem $P_{1-C}$ has a feasible solution only when $R_{\mathrm{th}}\leq \frac{\left(T-\tau_{\mathrm{ipt}}\right)f_{\mathrm{op}}}{KT}$.*

**Figure A1:**
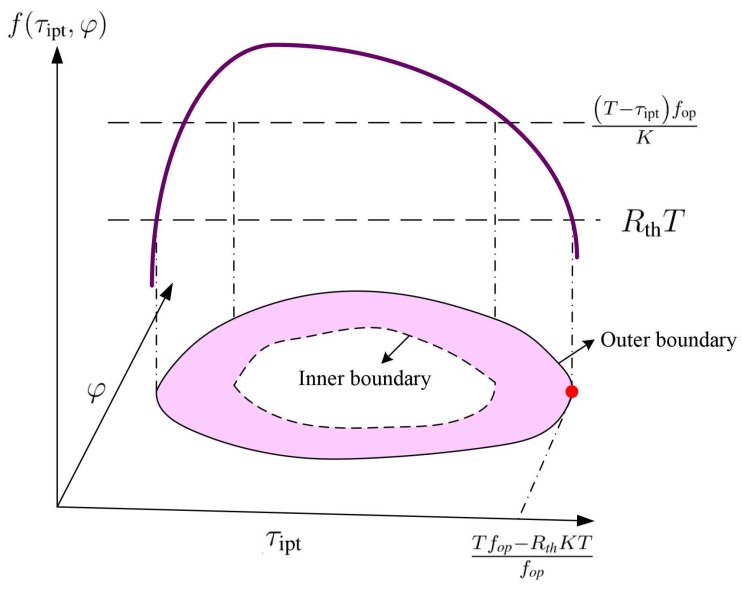
Illustration of the feasible solution set.

**Proof** **of** **Proposition** **1.** From Constraint (**C1**) and (**C2**) of Problem $P_{1-C}$, one can see that $R_{\mathrm{th}}T\leq f\left(\tau_{\mathrm{ipt}},\varphi \right)\leq \frac{\left(T-\tau_{\mathrm{ipt}}\right)f_{\mathrm{op}}}{K}$, i.e., $R_{\mathrm{th}}\leq \frac{\left(T-\tau_{\mathrm{ipt}}\right)f_{\mathrm{op}}}{KT}$. That is, when $R_{\mathrm{th}}\leq \frac{\left(T-\tau_{\mathrm{ipt}}\right)f_{\mathrm{op}}}{KT}$, the intersection set of the two constraints is not empty, which can be illustrated by Figure A1. Hence, Proposition 1 is proved. ☐

**Lemma** **1.** The optimal solution $\tau_{\mathrm{ipt}(\mathrm{local})}^{*}$ to Problem $P_{1-C}$ is $\tau_{\mathrm{ipt}(\mathrm{local})}^{*}=\frac{Tf_{\mathrm{op}}-R_{\mathrm{th}}KT}{f_{\mathrm{op}}}$.

**Proof** **of** **Lemma** **1.** The proof is shown in Appendix A. ☐

**Theorem** **1.** The optimal $\left\{\rho_{\left(\mathrm{local}\right)}^{*},\tau_{\mathrm{cpt}}^{*}\right\}$ of Problem $P_{1-C}$ is

(20) $$\left\{\begin{array}{cc} \rho_{\left(\mathrm{local}\right)}^{*} & =\frac{\sigma_{n}^{2}}{P_{\mathrm{AP}}{\left|h_{\mathrm{AP}-u}^{H}w\right|}^{2}}\left(2^{\frac{R_{\mathrm{th}}f_{\mathrm{op}}}{B\left(f_{\mathrm{op}}-KR_{\mathrm{th}}\right)}}-1\right),\\ \tau_{\mathrm{cpt}}^{*} & =\frac{KR_{\mathrm{th}}T}{f_{\mathrm{op}}}. \end{array}\right.$$

**Proof** **of** **Theorem** **1.** The proof is shown in Appendix B. ☐

### 3.2. Optimization of the Fog Offloading

For fog computing mode, the power minimization problem can be expressed as

(21)P2:minτipt,τu−f,ρ(offload),Pu−fEid+Eu−f−Eeh,s.t.C1:RAP−u≥Rth,C2:Ru−f≥RAP−u,C3:Pu−f≤Pu−f(max),C4:τipt+τu−f≤T,C5:τipt,τu−f∈(0,T),ρ(offload)∈(0,1).

Similar to the process of Problem $P_{1}$, let $\varpi ≜\tau_{\mathrm{ipt}}\rho_{\left(\mathrm{offload}\right)}$, $\lambda_{u-f}≜\tau_{u-f}P_{u-f}$, Problem $P_{2}$ is transformed as

(22)P2−A:minτipt,τu−f,ϖ,λu−fξBτiptlog1+ϖτiptPAPhAP−uHw2σn2+λu−f+ϖηPAPhAP−uHw2−τiptηPAPhAP−uHw2,s.t.C1:Bτiptlog1+ϖτiptPAPhAP−uHw2σn2≥RthT,C2:Bτu−flog1+λu−fτu−fhu−f2σs2≥Bτiptlog1+ϖτiptPAPhAP−uHw2σn2,C3:λu−fτu−f≤Pu−f(max),C4:τipt+τu−f≤T,C5:τipt,τu−f,∈(0,T),ρu−f∈[0,1].

The objective of Problem $P_{2-A}$ is a minimization of a concave function w.r.t $\tau_{\mathrm{ipt}}$, ϖ and $\lambda_{u-f}$. It is also difficult to solve due to the non-convexity of constraint sets (**C1**), (**C2**), and (**C3**).

**Proposition** **2.** *Problem $P_{2-A}$ has feasible solution only when $R_{\mathrm{th}}\leq B\frac{\tau_{u-f}}{T}\log\left(1+\frac{P_{u-f}^{\left(\max\right)}{\left|h_{u-f}\right|}^{2}}{\sigma_{s}^{2}}\right)$.*

**Proof** **of** **Proposition** **2.** From Constraint (**C1**) and (**C2**) of Problem $P_{2-A}$, one can see that when $R_{\mathrm{th}}\leq B\frac{\tau_{u-f}}{T}\log\left(1+\frac{P_{u-f}^{\left(\max\right)}{\left|h_{u-f}\right|}^{2}}{\sigma_{s}^{2}}\right)$, the intersection set of the two constraints is not empty. Hence, Proposition 2 is proved. ☐

Combined with the results above, and by using some mathematical manipulations, the objective function of Problem $P_{2-A}$ can be further expressed to be $\vartheta (\tau_{\mathrm{ipt}})$ as follows:

(23) $$\begin{array}{c} \vartheta \left(\tau_{\mathrm{ipt}}\right)=\xi R_{\mathrm{th}}T+\frac{\left(T-\tau_{\mathrm{ipt}}\right)\sigma_{s}^{2}}{{\left|h_{u-f}\right|}^{2}}\left(2^{\frac{R_{\mathrm{th}}T}{B(T-\tau_{\mathrm{ipt}})}}-1\right)-\tau_{\mathrm{ipt}}\eta \left(P_{\mathrm{AP}}{\left|h_{\mathrm{AP}-u}^{H}w\right|}^{2}-\sigma_{n}^{2}\left(2^{\frac{R_{\mathrm{th}}T}{B\tau_{\mathrm{ipt}}}}-1\right)\right), \end{array}$$

which is a convex function w.r.t $\tau_{\mathrm{ipt}}$, so Problem $P_{2-A}$ can be solved by using CVX tools [37]. Nevertheless, by using CVX tools, only numerical results can be obtained. In order to get some theoretically results and better understand the system, we further derive some semi-closed-form solutions to Problem $P_{2-A}$ as follows.

**Lemma** **2.** *Function ϑ($\tau_{\mathrm{ipt}}$) is convex w.r.t $\tau_{\mathrm{ipt}}$.*

**Proof** **of** **Lemma** **2.** By deriving the second-order deviation of function ϑ($\tau_{\mathrm{ipt}}$), it can be proved that it is always larger than zero, i.e., $\vartheta^{\prime \prime }\left(\tau_{\mathrm{ipt}}\right)=\frac{{\left(\ln2R_{\mathrm{th}}T\sigma_{s}\right)}^{2}}{B^{2}{\left|h_{u-f}\right|}^{2}{\left(T-\tau_{\mathrm{ipt}}\right)}^{3}}2^{\frac{R_{\mathrm{th}}T}{B(T-\tau_{\mathrm{ipt}})}}+\eta \frac{{\left(\ln2R_{\mathrm{th}}T\sigma_{n}\right)}^{2}}{\tau_{\mathrm{ipt}}^{3}}2^{\frac{R_{\mathrm{th}}T}{B\tau_{\mathrm{ipt}}}}>0$, which always holds for $\tau_{\mathrm{ipt}}\in \left(0,1\right)$. Therefore, Lemma 2 is proved. ☐

**Theorem** **2.** *The optimal solution to Problem $P_{2-C}$ is,*

(24) $$\left\{\begin{array}{cc} \tau_{\mathrm{ipt}(\mathrm{offload})}^{*} & =\{\tau_{\mathrm{ipt}}|\frac{\sigma_{s}^{2}}{{\left|h_{u-f}\right|}^{2}}\left(2^{\frac{R_{\mathrm{th}}T}{B\left(T-\tau_{\mathrm{ipt}}\right)}}\left(\ln2\frac{R_{\mathrm{th}}T}{B\left(T-\tau_{\mathrm{ipt}}\right)}-1\right)+1\right)\\ & =\eta P_{\mathrm{AP}}{\left|h_{\mathrm{AP}-u}^{H}w\right|}^{2}-\eta \sigma_{n}^{2}\left(2^{\frac{R_{\mathrm{th}}T}{B\tau_{\mathrm{ipt}}}}\left(1-\ln2\frac{R_{\mathrm{th}}T}{B\tau_{\mathrm{ipt}}}\right)-1\right),\tau_{\mathrm{ipt}}\in \left(0,T\right)\},\\ \rho_{\left(\mathrm{offload}\right)}^{*}\;\; & =\frac{\sigma_{n}^{2}}{P_{\mathrm{AP}}{\left|h_{\mathrm{AP}-u}^{H}w\right|}^{2}}\left(2^{\frac{R_{\mathrm{th}}T}{B\tau_{\mathrm{ipt}(\mathrm{offload})}^{*}}}-1\right),\\ \tau_{u-f}^{*}\;\;\;\;\;\; & =T-\tau_{\mathrm{ipt}(\mathrm{offload})}^{*},\\ P_{u-f}^{*}\;\;\;\;\;\; & =\frac{\sigma_{s}^{2}}{{\left|h_{u-f}\right|}^{2}}\left(2^{\frac{R_{\mathrm{th}}T}{B\left(T-\tau_{\mathrm{ipt}(\mathrm{offload})}^{*}\right)}}-1\right). \end{array}\right.$$

**Proof** **of** **Theorem** **2.** According to Lemma 2, $\tau_{\mathrm{ipt}(\mathrm{offload})}^{*}$ can be obtained by setting $\frac{\partial h(\tau_{\mathrm{ipt}(\mathrm{offload})})}{\partial \tau_{\mathrm{ipt}(\mathrm{offload})}}=0$, and its numerical result can be obtained by using the bisection method. Once $\tau_{\mathrm{ipt}(\mathrm{offload})}^{*}$ is obtained, by substituting it to the constraint equations, i.e., $B\tau_{\mathrm{ipt}}\log\left(1+\frac{\rho_{\left(\mathrm{offload}\right)}P_{\mathrm{AP}}{\left|h_{\mathrm{AP}-u}^{H}w\right|}^{2}}{\sigma_{n}^{2}}\right)=R_{\mathrm{th}}T$, $B\tau_{u-f}\log\left(1+\frac{{\left|h_{u-f}\right|}^{2}P_{u-f}}{\sigma_{s}^{2}}\right)=R_{\mathrm{th}}T$, and $\tau_{\mathrm{ipt}}+\tau_{u-f}=T$, respectively. The corresponding optimal $\rho_{\left(\mathrm{offload}\right)}^{*}$, $\tau_{u-f}^{*}$ and $P_{u-f}^{*}$ can be calculated, respectively. ☐

Here, we summarize the pseudocode of the procedure on how to calculate the optimal solution to the problem, which is shown in Algorithm 1.
**Algorithm 1. The optimal mode selection**1: Initialize system parameters such as *T*, $f_{\mathrm{op}}$, $R_{\mathrm{th}}$, *K*, *B*, $P_{\mathrm{AP}}$, $F_{0}$, α, $M_{c}$, $N_{0}$, ξ,2: Calculate optimal $\tau_{\mathrm{ipt}(\mathrm{local})}^{*}$ according to Lemma 1,3: Calculate optimal $\rho_{\left(\mathrm{local}\right)}^{*}$ and $\tau_{\mathrm{cpt}}^{*}$ according to Theorem 1,4: Calculate the minimal energy requirement (i.e., $E_{u(\mathrm{local})}$) by substituting $\tau_{\mathrm{ipt}(\mathrm{local})}^{*}$, $\rho_{\left(\mathrm{local}\right)}^{*}$, and $\tau_{\mathrm{cpt}}^{*}$ into the     objective function of Problem $P_{1}$,5: Calculate optimal $\tau_{\mathrm{ipt}(\mathrm{offload})}^{*}$ according to Theorem 2 by using the bisection method,6: Calculate optimal $\rho_{\left(\mathrm{offload}\right)}^{*}$, $\tau_{u-f}^{*}$, and $P_{u-f}^{*}$ based on $\tau_{\mathrm{ipt}(\mathrm{offload})}^{*}$ according to Theorem 1,7: Calculate the minimal energy requirement (i.e., $E_{u(\mathrm{offload})}$) by substituting $\tau_{\mathrm{ipt}(\mathrm{offload})}^{*}$, $\rho_{\left(\mathrm{offload}\right)}^{*}$, $\tau_{u-f}^{*}$, and $P_{u-f}^{*}$     into the objective function of Problem $P_{2}$,8: Compare $E_{u(\mathrm{local})}$ with $E_{u(\mathrm{offload})}$                If $E_{u(\mathrm{local})}\leq E_{u(\mathrm{offload})}$, then trigger the local computing mode,                Otherwise, trigger the fog computing mode.

## 4. Simulation Results

This section presents some simulation results to discuss the system performance of the two information processing modes. The system model shown in Figure 1 is simulated, where the HAP’s power $P_{\mathrm{AP}}$ is 1 watt and its transmitter number is set as $N_{A}=4$. The system bandwidth *B* = 2 MHz and noise power are $\sigma_{n}^{2}$ = $\sigma_{s}^{2}$ = −140 dBm. The Rice factor $K_{\mathrm{rice}}$ is 3.5 dB. Path-loss is modelled by the International Telecommunication Union (ITU) indoor channel model, i.e., $L=20\log f_{c}+n\log d-28$, with $f_{c}$ = 915 MHz and *N* = 22 [38]. Moreover, η=0.6 and ξ = $10^{-10}$ J/bit [35]. $R_{\mathrm{th}}$ = 20 Kb/s. $P_{u-f}^{\left(\max\right)}$ = $2\times 10^{-3}$ watt, $f_{\mathrm{op}}$ = $10^{9}$ operation/s, $M_{c}$ = $10^{4}$, α = 0.1, and $F_{0}$ = 3 [36]. In addition, $d_{\mathrm{AP}-u}=10$ m and $d_{u-f}=8$ m. Note that these parameters will not change unless otherwise specified. In this paper, all experiments are implemented over Mathworks Matlab R2017b ((Mathworks, Nedick, MA, USA) on a laptop equipped with 8.00 GHz Corei7-8550U CPU and 128 GB random access memory. Every point in the figures is the result averaged over 1000 independent channel realizations.

**Figure 3:**
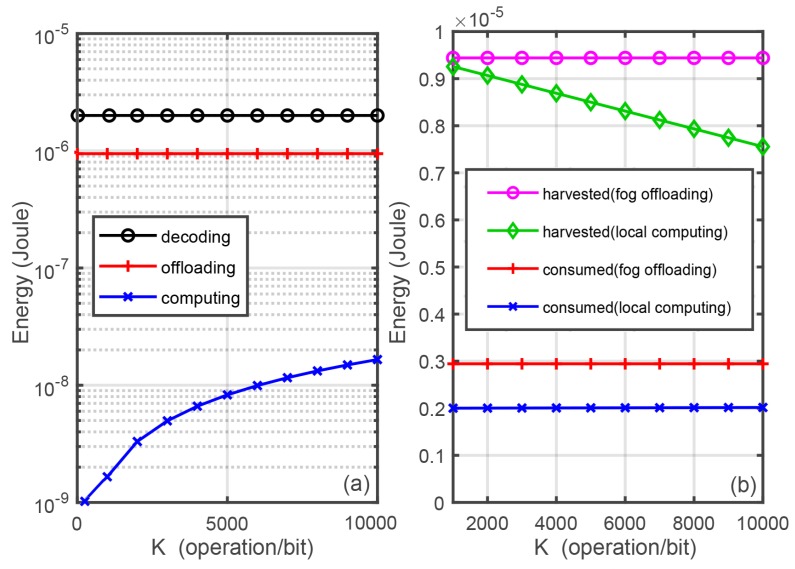
Harvested and required energy per frame versus *K*.

Figure 3 shows the harvested and required energy versus *K*. It can be seen that the energy required for decoding and offloading does not change with the increment of *K*, but that for computing changes linearly versus *K*. Moreover, the energy harvested in fog offloading mode is not change but that in the local computing mode is linearly deceasing versus *K* because it is affected by $\tau_{\mathrm{ipt}(\mathrm{local})}$ and $\rho_{\left(\mathrm{offload}\right)}$ that closely related to *K*, which is proportional to the energy requirement.

**Figure 4:**
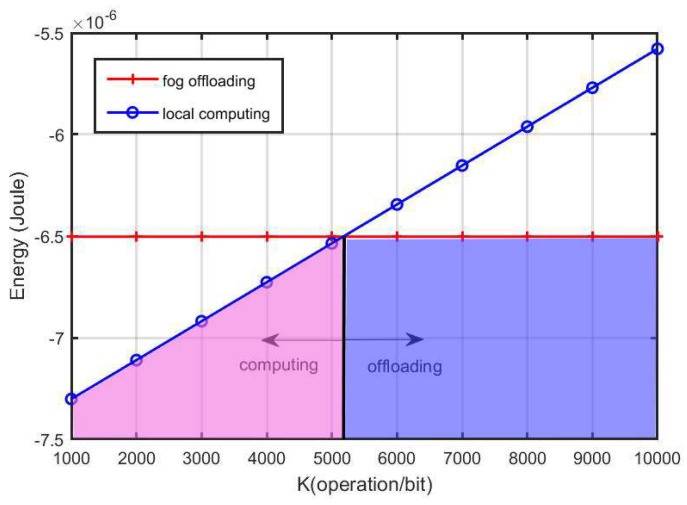
The minimal energy requirement of the two modes versus *K*.

Figure 4 plots the minimal power required by the local computing and fog offloading versus *K*. It can be seen that there is a intersection point, i.e., K= 5200, between the two curves associated with the two modes. As shown in the figure, when *K* is less than a certain value (i.e., the intersection point), the local computing is a better choice; otherwise, the fog computing is better.

**Figure 5:**
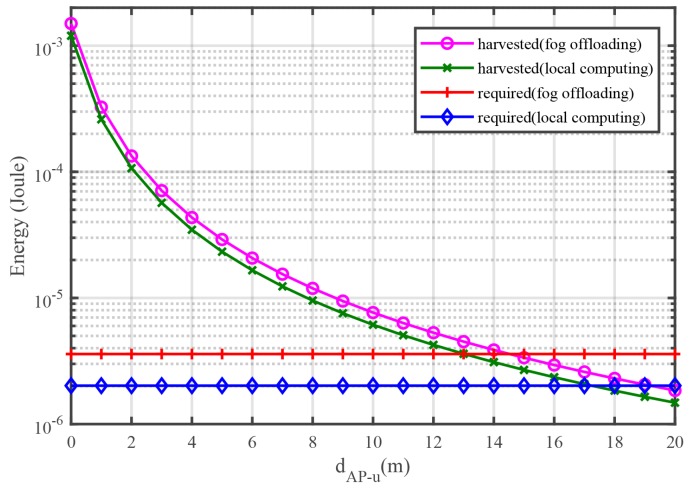
Harvested and required energy per frame versus $d_{\mathrm{AP}-u}$.

Figure 5 shows the harvested and required energy versus $d_{\mathrm{AP}-u}$, where the distance between the sensor and the FS is fixed, and the sensor is moved away from the HAP. One can see that with the increment of $d_{\mathrm{AP}-u}$, the required energy of the two modes almost does not change, but the harvested energy of both two modes is reduced with the increment of $d_{\mathrm{AP}-u}$ because the required energy is independent from $d_{\mathrm{AP}-u},$ but the energy harvesting closely depends on the path loss fading of the wireless channels.

**Figure 6:**
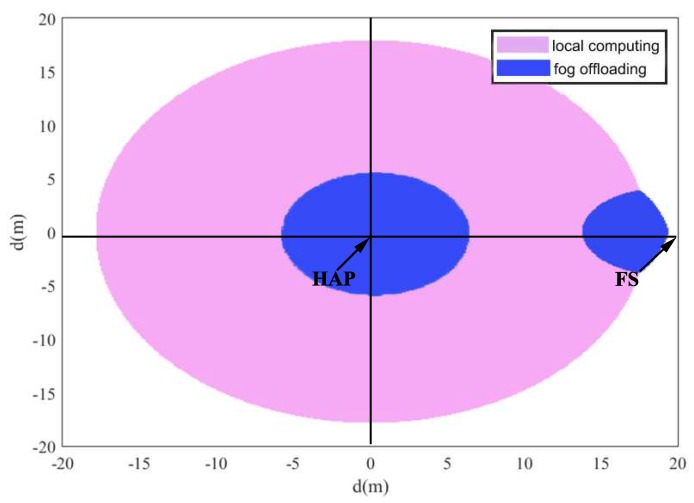
The mode selection deployment.

Motivated by the results in Figure 4 and Figure 5, the mode selection is discussed in Figure 6, where HAP is on the original point (i.e., (0,0)) of the coordinate system and the FS is positioned at the point with coordinate (0,20). The location of sensor is changed on the two-dimensional plane. We compare the minimal required energy of the two modes. It is shown that, in the blue area, fog offloading is a better choice and in the pink area, the local computing mode is a better choice. In the white region, the system has to firstly charge energy until the energy is sufficient to trigger any of the two modes.

**Figure 7:**
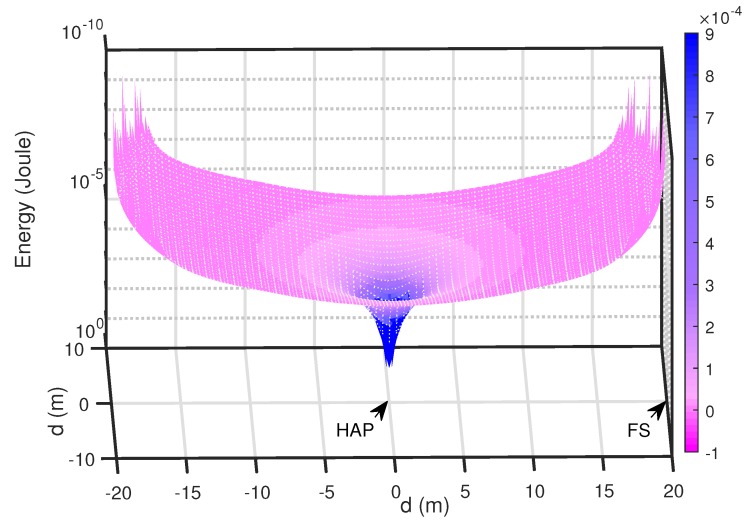
Energy requirements of selection mode.

The minimal required power of the sensor associated with the two modes is plotted in a 3D figure as shown in Figure 7. It can be seen that, when the sensor is closely positioned to the HAP, it requires a relatively low power to meet the information rate and computing requirements.

## 5. Conclusions

This paper studied a SWIPT-aware fog computing system with PS receiver architecture. Both local computing and fog offloading modes were investigated. The closed-form and semi-closed-form expressions for the optimal configurations were derived. Simulation results show that neither mode is always superior to the other one and there exists a threshold value, when the number of logic operations per bit associated with local computing is less than the threshold, the local computing is a better choice; otherwise, the fog offloading is better. In addition, the mode selection associated with the positions of the sensor for fixed HAP and the FS was also discussed, which shows that, when the sensor is close to the HAP or the FS, fog offloading mode is a better choice and, for the rest of the positions, local computing should be selected. 

## Figures and Tables

**Table 1:** The notation table.

Notations	Definations
*T*	the time length of transmission frame
$\tau_{\mathrm{ipt}(\mathrm{local})}$	the time length of energy harvesting in the local computing mode
$\tau_{\mathrm{ipt}(\mathrm{offload})}$	the time length of energy harvesting in the fog offloading mode
$\tau_{\mathrm{cpt}}$	the time used for local computing
$\tau_{u-f}$	the time used for task offloading from the sensor to the FS
$P_{\mathrm{AP}}$	the transmit power of the HAP
$N_{A}$	the number of antennas at the HAP
*s*	the RF signal symbol transmitted by the HAP
w	the beamforming vector
$\sigma_{n}^{2}$	the noise received at the receiver
$h_{\mathrm{AP}-u}$	the complex channel vector from the HAP to the sensor
$\rho_{\left(\mathrm{local}\right)}$	the power splitting factor in the local computing mode
$\rho_{\left(\mathrm{offload}\right)}$	the power splitting factor in the fog offloading mode
η	the energy conversion efficiency of the EH circuit
$R_{\mathrm{AP}-u}$	the achievable information rate at the sensor
*B*	the system frequency bandwidth
ξ	the energy requirement for decoding per bit
$R_{u-f}$	the achievable information rate associated with the offloading
$P_{u-f}$	the transmit power at the sensor
$\sigma_{s}^{2}$	the receiver’s noise power
$P_{u-f}^{\left(\max\right)}$	the maximal available transmit power
*K*	the number of logic operations per bit
$h_{u-f}$	the complex-valued channel coefficient from the sensor to the FS
$R_{\mathrm{th}}$	the minimum information transmission rate requirement from the HAP to the sensor
$f_{\mathrm{op}}$	the maximum number of the operations per second at the sensor
$E_{\mathrm{eh}}$	the harvested energy at the sensor
$E_{\mathrm{id}}$	the required energy for information decoding at the sensor
$E_{\mathrm{cpt}}$	the local computing energy requirement
$E_{u-f}$	the energy required for task offloading at the sensor
$E_{u}$	the total required energy at the sensor

## Abbreviations

The following abbreviations are used in this manuscript:

IoT	Internet of Things
MEC	mobile edge computing
WSN	wireless sensor networks
WPAN	wireless personal area networks
RF	radio frequency
SWIPT	simultaneous wireless information and power transfer
HAP	hybrid access point
FS	fog server
TS	time switching
PS	power splitting
TDMA	time division multiple access
FDMA	frequency division multiple access
CDMA	code division multiple access
CSI	channel state information
MISO	multiple input single output
MRT	maximum rate transmission

## Appendix A

The objective function of Problem $P_{1-C}$, i.e., $F(\tau_{\mathrm{ipt}},\varphi )$, is a concave function, so the minimal value of this concave function must be at the outer boundary of its feasible set. Moreover, the left-hand side of Constraint (**C1**) and (**C2**) of Problem $P_{1-C}$ are convex, so that the outer boundary of the feasible set of Problem $P_{1-C}$ satisfies that $f\left(\tau_{\mathrm{ipt}},\varphi \right)=R_{\mathrm{th}}T$, which is presented by the black line on the $\left(\tau_{\mathrm{ipt}},f\left(\tau_{\mathrm{ipt}},\varphi \right)\right)$ plane as shown in Figure A1. When the points on the binary, i.e., $\left\{\tau_{\mathrm{ipt}},\varphi \right\}$ satisfies $f\left(\tau_{\mathrm{ipt}},\varphi \right)=R_{\mathrm{th}}T$, $F(\tau_{\mathrm{ipt}},\varphi )$ achieves its minimal value. This is equivalent to the minimal value of the $F(\tau_{\mathrm{ipt}},\varphi )$ being obtained when $B\tau_{\mathrm{ipt}}\log\left(1+\frac{\varphi P_{\mathrm{AP}}{\left|h_{\mathrm{AP}-u}^{H}w\right|}^{2}}{\tau_{\mathrm{ipt}}\sigma_{n}^{2}}\right)=R_{\mathrm{th}}T$. Therefore, the objective function of Problem $P_{1-C}$ can be transformed into

(A1) $$\begin{array}{c} F\left(\tau_{\mathrm{ipt}},\varphi \right)=C_{1}R_{\mathrm{th}}T-C_{2}\left(\tau_{\mathrm{ipt}}-\varphi \right). \end{array}$$

Then, the suitable $\left\{\tau_{\mathrm{ipt}},\varphi \right\}$ at the outer boundary $f\left(\tau_{\mathrm{ipt}},\varphi \right)=R_{\mathrm{th}}T$ has to be determined to make $F(\tau_{\mathrm{ipt}},\varphi )$ reach the minimal value. According to the boundary condition $B\tau_{\mathrm{ipt}}$ $\log\left(1+\frac{\varphi P_{\mathrm{AP}}{\left|h_{\mathrm{AP}-u}^{H}w\right|}^{2}}{\tau_{\mathrm{ipt}}\sigma_{n}^{2}}\right)=R_{\mathrm{th}}T$ and Equation (A1), one can obtain that $\varphi =\frac{\tau_{\mathrm{ipt}}\sigma_{n}^{2}}{P_{\mathrm{AP}}{\left|h_{\mathrm{AP}-u}^{H}w\right|}^{2}}\left(2^{\frac{R_{\mathrm{th}}T}{B\tau_{\mathrm{ipt}}}}-1\right)$. Then, by substituting it into the objective function, the objective function is transformed into

(A2) $$\begin{array}{cc} g(\tau_{\mathrm{ipt}}) & =C_{1}R_{\mathrm{th}}T\underset{\left(b\right)}{\underset{︸}{-C_{2}\tau_{\mathrm{ipt}}\left(1-\frac{\sigma_{n}^{2}}{P_{\mathrm{AP}}{\left|{h_{\mathrm{AP}-u}}^{H}w\right|}^{2}}{\overset{︷}{\left(2^{\frac{R_{\mathrm{th}}T}{B\tau_{\mathrm{ipt}}}}-1\right)}}^{\left(a\right)}\right)}}. \end{array}$$

$g(\tau_{\mathrm{ipt}})$ is a monotonically decreasing function w.r.t the first constant term but a monotonically increasing function w.r.t. the second term. Nevertheless, in the second term, the increasing rate w.r.t $\tau_{\mathrm{ipt}}$ of (b) part in Equation (A2) is smaller than the increasing rate w.r.t $\tau_{\mathrm{ipt}}$ of (a) in Equation (A2). Thus, $g(\tau_{\mathrm{ipt}})$ is a decreasing function w.r.t $\tau_{\mathrm{ipt}}$. As a result, when $\tau_{\mathrm{ipt}}$ reach the upper boundary, i.e., $\tau_{\mathrm{ipt}}=\frac{Tf_{\mathrm{op}}-R_{\mathrm{th}}KT}{f_{\mathrm{op}}}$, $g(\tau_{\mathrm{ipt}})$ arrives at its minimal value. Note that, as shown in Figure A1, $\tau_{\mathrm{ipt}}=\frac{Tf_{\mathrm{op}}-R_{\mathrm{th}}KT}{f_{\mathrm{op}}}$ is obtained in the case that the inequality of Constraint (**C1**) and (**C2**) of Problem $P_{1-C}$ adopts equal signs simultaneously, namely, the dynamic inner boundary meets the outer boundary $\frac{\left(T-\tau_{\mathrm{ipt}}\right)f_{\mathrm{op}}}{K}=R_{\mathrm{th}}T$. Therefore, Lemma 1 is proved.

## Appendix B

Following Lemma 1, when $\left\{\tau_{\mathrm{ipt}},\varphi \right\}$ is at the outer boundary $f\left(\tau_{\mathrm{ipt}},\varphi \right)=R_{\mathrm{th}}T$, the objective function $F(\tau_{\mathrm{ipt}},\varphi )$ achieves its minimal value. $\varphi =\frac{\tau_{\mathrm{ipt}}\sigma_{n}^{2}}{P_{\mathrm{AP}}{\left|h_{\mathrm{AP}-u}^{H}w\right|}^{2}}\left(2^{\frac{R_{\mathrm{th}}T}{B\tau_{\mathrm{ipt}}}}-1\right)$ and $\tau_{\mathrm{ipt}}$ = $\frac{Tf_{\mathrm{op}}-R_{\mathrm{th}}KT}{f_{\mathrm{op}}}$, combined with $\rho =\frac{\varphi }{\tau_{\mathrm{ipt}}}$, we have that $\rho_{\left(\mathrm{local}\right)}^{*}$ = $\frac{\sigma_{n}^{2}}{P_{\mathrm{AP}}{\left|h_{\mathrm{AP}-u}^{H}w\right|}^{2}}\left(2^{\frac{R_{\mathrm{th}}f_{\mathrm{op}}}{B\left(f_{\mathrm{op}}-KR_{\mathrm{th}}\right)}}-1\right)$. Moreover, when $\tau_{\mathrm{cpt}}<\frac{KR_{\mathrm{th}}T}{f_{\mathrm{op}}}$, $F(\tau_{\mathrm{ipt}},\varphi )$ has a smaller value with a larger $\tau_{\mathrm{ipt}}$. Thus, $\tau_{\mathrm{cpt}}^{*}=\frac{KR_{\mathrm{th}}T}{f_{\mathrm{op}}}$. Therefore, Theorem 1 is proved.
